# Respiratory virus-induced bacterial dysregulation in pediatric airway tissue and the dual actions of Echinacea in reducing complications

**DOI:** 10.3389/fphar.2025.1579551

**Published:** 2025-05-30

**Authors:** Selvarani Vimalanathan, Mahfuza Sreya, Ranganayaki Nandanavanam, Roland Schoop, Giuseppe Gancitano, Saba Saberi, Anna Malikovskaia, James Hudson

**Affiliations:** ^1^ Pathology and Laboratory Medicine, University of British Columbia, Vancouver, BC, Canada; ^2^ Women+ and Children’s Health Sciences, University of British Columbia, Vancouver, BC, Canada; ^3^ Applied Biology, University of British Columbia, Vancouver, BC, Canada; ^4^ Medical Department, A.Vogel AG, Roggwil, Switzerland; ^5^ Department of Experimental and Clinical Medicine, University of Florence, Florence, Italy

**Keywords:** pediatrics, EpiAirway viral-bacterial superinfections, *Streptococcus pneumoniae*, *Haemophilus influenzae* type b, respiratory syncytial virus, human parainfluenza virus type 3, rhinovirus, *Echinacea purpurea*

## Abstract

**Introduction:**

Respiratory tract infections (RTIs) contribute to pediatric morbidity and are often complicated by viral-bacterial superinfections, which exacerbate disease severity and increase antibiotic use. This study examined viral-induced bacterial adhesion in an *ex vivo* pediatric airway model and the therapeutic potential of *Echinacea purpurea* extract.

**Methods:**

EpiAirway tissue from a 6-year-old boy was infected with respiratory syncytial virus (RSV), human parainfluenza virus type 3 (HPIV3), or rhinovirus 14 (RV14). Adhesion of *Haemophilus influenzae* type b (Hib) and *Streptococcus pneumoniae* (*S. pneumoniae*) was assessed alongside the expression of platelet-activating factor receptor (PAFr), intercellular adhesion molecule-1 (ICAM-1), and carcinoembryonic antigen-related cell adhesion molecule-1 (CEACAM-1). Echinaforce^®^ (EF extract) was tested for its effect on bacterial dysregulation.

**Results:**

RSV and HPIV3 increased bacterial adhesion by upregulating PAFr, ICAM-1, and CEACAM-1. Hib adhered primarily via ICAM-1, while *S. pneumoniae* favored PAFr. RV14 strongly induced CEACAM-1 but did not cause significant bacterial dysregulation. EF significantly reduced virus-induced receptor overexpression, resulting in inhibition of bacterial adhesion and biofilm-like formation.

**Conclusion:**

Our findings provide a mechanistic explanation for the observed effects of *E. purpurea* in reducing RTI complications and the need for antibiotic prescriptions in clinical settings.

## 1 Introduction

Respiratory tract infections (RTIs) remain the leading cause of antibiotic prescriptions, despite international efforts promoting their judicious use (Llor and Bjerrum, 2014; Cars et al., 2017; Diseases et al., 2018). Over 90% of RTIs are viral in origin but may exacerbate into viral-bacterial superinfections clinically manifesting as tonsillitis, sinusitis, otitis media, or even pneumonia (Aitken and Taylor, 1998; Fendrick et al., 2003; Del Mar 2016). Children are particularly vulnerable to RTIs, due to their developing immune systems and frequent exposure to respiratory pathogens, which account for the single major reason for pediatric healthcare visits (60%). Up to 75% of antibiotic prescriptions are attributed to these infections (Kristjansson et al., 2005; Message and Johnston, 2009; Liao et al., 2011; van Houten et al., 2019; Zhou et al., 2022; Dharanindra et al., 2023; Picca et al., 2023; Igirikwayo et al., 2024). Factors such as fear of worsening conditions, cultural expectations, and the widespread availability of prescription-free antibiotics (especially online) undermine efforts to reduce unnecessary antibiotic use and propel the development of antimicrobial resistance (AMR) (Mangione-Smith et al., 1999; Morgan et al., 2011; Auta et al., 2019; Lebanova et al., 2023).

Various mechanisms have been proposed to explain virus-induced bacterial superinfections. In addition to epithelial damage and dampened mucociliary clearance, viruses can upregulate the expression of bacteria-binding receptors such as platelet-activating factor receptor (PAFr), intercellular adhesion molecule-1 (ICAM-1), and carcinoembryonic antigen-related cell adhesion molecule-1 (CEACAM-1). Consequently, bacterial attraction to the site of viral infection and involvement in the infection process is induced (McCullers, 2006; Avadhanula et al., 2007; Bosch et al., 2013; Siegel et al., 2014; Wu et al., 2021). Recent literature indicates that not all respiratory viruses exhibit the same potential for complications and that different bacteria employ distinct receptor-binding patterns, resulting in a rather discrete regulation of bacterial adherence. For example, *Streptococcus pneumoniae* (*S. pneumoniae*) demonstrates a high affinity to PAFr and ICAM-1 rather than CEACAM-1, whereas *Haemophilus influenzae* type b (Hib) additionally utilizes CEACAM-1 receptor for binding to epithelial cells (Ishizuka et al., 2001; Avadhanula et al., 2007; O’Toole et al., 2016).

Understanding the versatile pathological effects of viruses that predispose airway tissue to bacteria colonization may pave the way for development of effective therapeutics against RTI complications, thereby harbouring the potential to reduce the need for antibiotics. Effective management of RTIs in children is of utmost importance, as antibiotic overuse in this population is a major contributor to antibiotic resistance (Liao et al., 2011; Moreno, 2013; Cars et al., 2017; Sur and Plesa, 2022).

A proprietary *Echinacea purpurea* extract (Echinaforce^®^ [EF] extract) has recently demonstrated a remarkable reduction in antibiotic prescriptions by up to 76% through the prevention of RTIs and their complications (Ogal et al., 2021). Additionally, a meta-analysis of 30 clinical studies involving 5,652 participants confirmed that Echinacea reduced viral respiratory infections by 32% but the progression to secondary complications, and the subsequent need for antibiotics by up to 56% and 71%, respectively (*p* < 0.05). The RTI preventive benefits were primarily attributed to Echinacea’s antiviral effects, with multiple phytochemical compounds shown to interfere with pathogen ligand-host receptor interactions. Anti-inflammatory and immune-modulatory actions were revealed under *in vitro* and *ex vivo* conditions to suppress virus pathology and to beneficially impact patient’s recovery, whereas evidence on direct bactericidal and/or antibacterial effects remains unclear. All pharmacological actions described so far could not sufficiently explain the ascending effects from viral prevention to complications and, finally, antibiotic use and why effects in pediatrics were strongest (Pleschka et al., 2009; Nicolussi et al., 2022; Gancitano et al., 2024).

The objective of this study was to further explore pathophysiological interactions between respiratory viruses and bacteria specifically in pediatric airway tissue, and to investigate the potential of EF to mitigate virus-induced bacterial dysregulation. Using a combination of primary epithelial cells and a physiologically relevant *ex vivo* model, we examined the effects of EF on bacterial adhesion and host receptor modulation following viral infection. We first used primary airway epithelial cells derived from a 6-year-old child to investigate the effects of three clinically relevant respiratory viruses: rhinovirus type 14 (RV14), human parainfluenza virus type 3 (HPIV3), and respiratory syncytial virus (RSV) focusing on host receptor expression and subsequent bacterial adherence. We evaluated bacterial adherence using *S. pneumoniae* serotype 19F and *H. influenzae* type b (Hib), two major pediatric pathogens selected for their roles in invasive diseases such as pneumonia, otitis media, and tonsilitis. These strains were also selected due to their increasing resistance to commonly used beta-lactam antibiotics: *S. pneumoniae* 19F and Hib (de Groot et al., 1991; Mavroidi et al., 2007; Mayanskiy et al., 2014; Lyu et al., 2017; Ekinci et al., 2021; Lister et al., 2021; Oliveira et al., 2021). Despite widespread vaccination, both pathogens remain clinically significant in children, underscoring the need for alternative preventive strategies.

To expand upon these findings in a more physiologically relevant setting, we developed a novel air-liquid interface (ALI) model using epithelial cells derived from the same pediatric donor. This three-dimensional culture system (EpiAirway) recapitulates the pseudostratified airway epithelium, including ciliated cells, mucus-producing goblet cells, and tight junctions, thereby mimicking the multilayered structure of the respiratory tract *in vivo* (Dvorak et al., 2011; Michi and Proud, 2021; Lee et al., 2023; Silva et al., 2023). Compared to traditional animal models, which often fail to replicate the human airway environment due to differences in epithelial architecture and immune signaling, the ALI model offers a more reliable and suitable system for studying human respiratory infections. This is particularly relevant for RSV, which exhibits limited replication and altered pathology in rodents due to species-specific receptor expression and immune responses (Rijsbergen et al., 2021; Ito et al., 2023). The ALI model enabled a more detailed investigation of RSV-induced receptor modulation and subsequent bacterial adherence and the effect of EF under near *in vivo* conditions. All viruses and bacterial strains used were clinical isolates, except for RV14.

## 2 Materials and methods

### 2.1 Experimental models and procedures for studying viral-bacterial interactions

#### 2.1.1 NHBE cells and EpiAirway tissue

Normal Human Bronchial Epithelial (NHBE) cells and EpiAirway 3D tissue models (AIR 100-D2) were obtained from MatTek Corporation (Ashland, MA, United States). Both were derived from the same donor, a 6-year-old Caucasian child with no airway disease, ensuring consistency. The EpiAirway model, composed of tracheal/bronchial epithelial cells, forms a pseudostratified epithelium with goblet, ciliated, and basal cells, mimicking the respiratory epithelium.

EpiAirway tissues were cultured and maintained following the manufacturer’s protocol. Briefly, 2-week-old tissues were received and, upon arrival, removed from the packaging, placed in a 6-well plate containing 1 mL of maintenance medium (AIR 100-MM), and equilibrated at 37°C with 5% CO_2_ for 16 h. The tissues were then transferred to 6-well trays (HNG-TOP-6, MatTek) and supplemented basolaterally with 8 mL of maintenance medium (AIR100-MM). The tissues were further matured in-house for 7 days before infection, with 8 mL of basolateral medium replaced every 48 h and mucus washes (400 µL of 1X PBS on the apical side) performed every 3–4 days during maturation and prior to infection.

NHBE cells were cultured in NHBE growth medium (NHBE-GM) at 37°C, subcultured to passage 3, and seeded to grow to 70%–80% confluence.

#### 2.1.2 Virus and bacteria cultures

The H-1 sub-clone of HeLa cells, HEp-2 cells, and monkey kidney (LLC-MK2) cells were all originally obtained from the American Type Culture Collection (ATCC), Rockville, MD. These cultures were maintained in Dulbecco’s Modified Eagle Medium (DMEM) in cell culture flasks, supplemented with 5%–10% fetal bovine serum, and incubated at 37°C in a 5% CO_2_ atmosphere. The exception was the LLC-MK2 cells, which were grown in ATCC 199 medium supplemented with 10% horse serum. No antibiotics or antimycotic agents were used in the cultures.

Human parainfluenza virus type 3 (HPIV3) was obtained from the BC Centre for Disease Control, Vancouver, Canada, and propagated in LLC-MK2 cells.

Respiratory syncytial virus (RSV) strain A2 (rgRSV3031) was provided by Dr. Turvey, University of British Columbia, and propagated in HEp-2 cells.

Rhinovirus type 14 (RV14) was sourced from ATCC and propagated in H-1 cells at 33°C. Viral stocks were clarified by centrifugation, filtered through 0.22 µm membranes, and stored at −80°C. Viral titers were determined using plaque assays, yielding concentrations of 10^5^ to 10^7^ plaque-forming units (PFU)/mL.

Based on preliminary experiments, RV14 was selected for this study as it induced significant bacterial adhesion without excessive cytopathic effects at an MOI of 1.

Bacteria *S. pneumoniae* (serotype 19F) and *H. influenzae* type b (Hib) were obtained from the Clinical Microbiology Proficiency Testing Lab, University of British Columbia. *S. pneumoniae* was cultured on 5% sheep blood agar, and Hib on chocolate agar (Hardy Diagnostics, Santa Maria, CA, USA). Overnight cultures were prepared in the recommended liquid media, washed twice in PBS, and adjusted to 1 × 10^8^ CFU/mL in cell growth media for infection.

### 2.2 Standardized *Echinacea purpurea* preparation

Echinaforce^®^ (EF extract supplied by A. Vogel AG, Switzerland) is a 65% ethanolic extract from 95% freshly harvested aerial parts and 5% roots of *E. purpurea*. The phytochemical characterization of Echinaforce has been described in detail elsewhere (Sharma et al., 2009).

### 2.3 Cytotoxicity assays

The cytotoxicity of EF in EpiAirway tissues was evaluated using an MTT assay kit (Part No. MTT-100) from MatTek Life Sciences, in accordance with the manufacturer’s protocol. Briefly, the mucus was removed from the apical side of the tissues, which were then exposed to basal media containing EF at concentrations of 1:200 or 1:400. Vehicle controls containing 0.325% v/v ethanol (matching the ethanol content in a 1:200 EF dilution) were also included. The tissues were cultured for 72 h, after which the MTT assay was performed.

For assessing the cytotoxicity of EF on NHBE cells, an MTS assay (Promega, Madison, WI) was conducted following a previously described method (Vimalanathan et al., 2017). NHBE cells were seeded into 96-well culture plates and allowed to form monolayers over a 24-h period. The medium was then replaced with NHBE maintenance medium containing EF at concentrations ranging from 1:100 to 1:600, or with control medium. The cells were cultured for 72 h, after which the MTS assay was performed to calculate the number of viable cells.

Throughout this study, all infected and uninfected controls were maintained with 0.325% ethanol (v/v).

### 2.4 Virus infection and bacterial adhesion assay on EpiAirway tissues and NHBE cells

#### 2.4.1 Virus infection protocol on EpiAirway tissues

Prior to RSV infection, the apical surfaces of EpiAirway tissues were rinsed with DPBS to remove mucus and transferred to 12-well plates containing 0.5 mL of tissue maintenance medium per well. RSV was diluted in 100 µL of EpiAirway medium (MOI 1) and applied apically, while uninfected controls received medium only. After a 2-h incubation at 37°C, unbound virus was removed with two DPBS washes, and the tissues were transferred to 6-well trays (HNG-TOP-6, MatTek). EF was added basolaterally with the medium at 1:200 and 1:400 dilutions (40–80 μg/mL), while control groups received medium containing 0.325% ethanol. The tissues were then incubated at 37°C in a CO_2_ incubator for 72 h.

#### 2.4.2 Bacterial adhesion assay on EpiAirway tissues

For bacterial infections, *S. pneumoniae* and Hib were cultured overnight, washed, resuspended to 1 × 10^8^ CFU/mL, and inoculated apically (MOI 10–12). After 2 h at 37°C, unbound bacteria were removed by five DPBS washes, and the tissues were fixed with 10% formalin for 45 min. Adherent bacteria were quantified using specific primary antibodies: NB100-64570 for *S. pneumoniae* (Novus Biologicals, United States) and orb157421 for Hib (Biorbyt, United States).

To simulate the *in vivo* airway environment, we utilized a physiologically relevant air-liquid interface (ALI) culture system. This model allowed EF extract to be applied to the basal side, mimicking systemic absorption, while viral infections and bacterial adhesion occurred on the apical side. The ALI setup enabled us to study the interplay between viruses, bacterial adhesion, and receptor expression.

#### 2.4.3 Virus infection protocol on NHBE cells

The viral infection and bacterial adhesion assays on NHBE cells were adapted from a previously described protocol (Vimalanathan et al., 2017). NHBE cells (1 × 10^5^ per well) were cultured in 12-well plates to 80%–90% confluence. For viral infection, cells were exposed to RSV (MOI 1.0 PFU/cell), RV14 (2.5 PFU/cell), or HPIV3 (0.1 PFU/cell) and incubated at 37°C for 1 h. After removing unabsorbed virus with DPBS, EF extract was added at final dilutions of 1:200 or 1:400, while control cells received medium containing 0.325% ethanol. Cells infected with RV14 were incubated at 33°C, and those infected with RSV or HPIV3 were maintained at 37°C, all for 72 h in 5% CO_2_.

#### 2.4.4 Bacterial adhesion assay on NHBE cells

Bacterial adhesion assays were subsequently performed on virus-infected NHBE cells. *S. pneumoniae* or Hib, at an MOI of 10, was added to the apical surface of infected cells and incubated for 2 h at 37°C. Non-adherent bacteria were removed with five washes of DPBS. Cells with adhered bacteria were detached using 0.025% trypsin-EDTA, serially diluted, and plated on 5% sheep blood or chocolate agar for bacterial colony enumeration. Plates were incubated at 37°C with 5% CO_2_. Cell counts were determined using a cytometer, and bacterial adhesion was normalized to cell numbers. All assays were performed in triplicate and repeated three times.

### 2.5 Receptor blocking and immunohistochemical analysis

#### 2.5.1 Antibody blocking of bacterial adhesion

These procedures were performed as previously described (Vimalanathan et al., 2017). Briefly, cells infected with RSV, RV14, and HPIV3 were cultured for 72 h, then blocked for ICAM-1, PAFr, and CEACAM-1 receptors by adding the following antibodies: mouse monoclonal antibody to ICAM-1 (Abcam, United States), rabbit polyclonal antibody to PAFr, and rabbit monoclonal antibody to CEACAM-1, each at a concentration of 5 μg/mL. The cells were incubated with these antibodies for 2 h at 37°C. After blocking, the cells were washed and incubated with S. *pneumoniae* or Hib for 2 h at 37°C, and the bacterial adhesion assay was conducted as described previously.

#### 2.5.2 EpiAirway tissue: immunohistochemical analysis

Immunohistochemical staining for *S. pneumoniae* and Hib was performed on 5 µm paraffin sections as per manufacturer’s protocols. Sections were deparaffinized, rehydrated, and subjected to heat-induced antigen retrieval using sodium citrate buffer (pH 6.0 or pH 5.0) at 95°C–98°C for 10 min, followed by cooling and washing. After permeabilization with 0.3% Triton X-100 and blocking with 3% BSA in PBST, sections were incubated overnight at 4°C with the primary antibodies: anti-*S. pneumoniae* (10 μg/mL, NB100-64570, Novus Biologicals) and anti-Hib (10 μg/mL, orb157421, Biorbyt). Negative controls used antibody dilution buffer without primary antibodies.

Following incubation, sections were washed and incubated for 1 h at room temperature with secondary antibodies: DayLight 488 Rabbit anti-Mouse IgG (NBP1-75251, Novus Biologicals) for *S. pneumoniae* and Alexa Fluor^®^ 647 Donkey anti-Rabbit IgG (ab150075, Abcam) for Hib. After washing, sections were mounted with Fluoroshield Mounting Medium containing DAPI. Images were captured using a Zeiss Axio Observer Z1 fluorescent microscope and analyzed with AxioVision 4.8 and ImageJ software for mean fluorescence intensity.

#### 2.5.3 Immunocytochemistry and confocal imaging of NHBE cells

NHBE cells were cultured on glass slides in 24-well trays at a density of 1 × 10^4^ cells per well. After 24 h, the cells were infected with RSV, RV14, or HPIV3 as previously described (Vimalanathan et al., 2017). Immunostaining was performed 72 h post-infection. Cells were fixed with 1% paraformaldehyde for 15 min at room temperature (RT), washed twice with cold PBS, and permeabilized with 0.5% saponin in PBS (Sigma Aldrich, St. Louis, MO) for 10 min at RT. Non-specific binding was blocked with 1% BSA (Cedarlane, Burlington, ON, Canada) in PBS for 1 h at RT.

Primary antibodies, including mouse monoclonal anti-ICAM-1 (ab2213, Abcam, Cambridge, MA, United States), rabbit polyclonal anti-PAFr (ab104162, Abcam), and rabbit polyclonal anti-CEACAM-1 (ab108397, Abcam), were diluted to 5 μg/mL in 1% BSA with 0.1% Tween 20 and incubated with the cells overnight at 4°C. Following primary antibody incubation, cells were washed with PBS and treated with secondary antibodies, Rabbit anti-Mouse IgG DyLight 488 (NBP1-75251, Novus Biologicals, CO, United States) for ICAM-1 and Goat anti-Rabbit IgG Alexa Fluor 594 (ab150080, Abcam) for PAFr and CEACAM-1, diluted 1:500 in 1% BSA for 1 h at RT in the dark.

After washing with PBS, fluorescence imaging was performed using a Zeiss Axio Observer Z1 inverted fluorescence microscope with a 20× lens. Images were analyzed using Zeiss AxioVision Software 4.8, and mean fluorescence intensity was calculated using ImageJ.

### 2.6 Statistical analysis

Statistical analyses were performed using GraphPad Prism 10 (version 10.4.0). Ordinary one-way analysis of variance (ANOVA) was conducted, followed by Dunnett’s *post hoc* test for multiple comparisons. Data are presented as mean ± standard deviation (SD) or standard error of the mean (SEM), as specified in the Figure legends. Statistical significance was defined as *p < 0.05.

## 3 Results

### 3.1 Cell viability in EpiAirway tissues and NHBE cells

We initially determined that EF extract (Echinaforce^®^), in dilutions ranging from 1:200 to 1:1600, was not cytotoxic to 3D EpiAirway tissues or primary NHBE cell cultures following exposure for up to 72 h, as assessed by MTT and MTS assays. Even at a highest tested concentration, corresponding toa 1:200 dilution, 80 μg/mL of EF extract dry mass, no cytotoxic effects were observed in eithercell model.After 72 h of exposure to NHBE cells or EpiAirway tissue, EF at a 1:200 dilution resulted in 97% and 98% cell viability, respectively. Meanwhile, dilutions ranging from 1:400 to 1:600 or higher maintained 100% cell viability, comparable to untreated controls.

### 3.2 Effects of viral infections and EF on bacterial dysregulation and receptor expression

#### 3.2.1 RSV-induced bacterial adhesion in EpiAirway tissues

**FIGURE 1:**
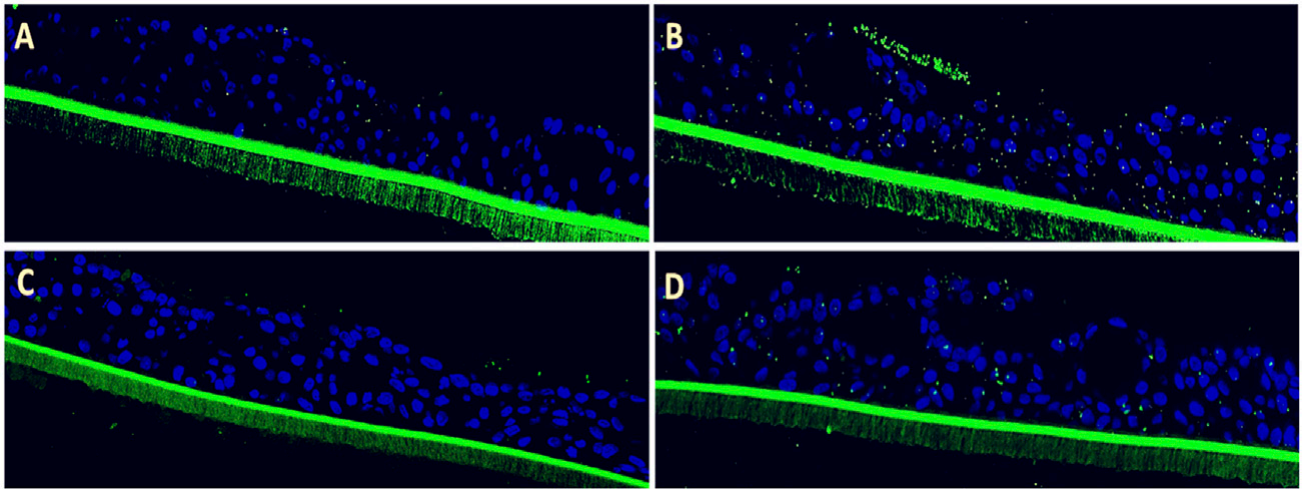
Efficacy of Echinaforce in reducing RSV-induced *S. pneumoniae* adhesion in pediatric EpiAirway tissue. **(A)** EpiAirway tissues cultured in an air-liquid interface (ALI) were stained with anti-*S. pneumoniae* antibody (green) and DAPI for nuclei, visualized at ×20 magnification. Representative images are shown for the following conditions: **(A)** Vehicle Control + *S. pneumoniae*, **(B)** RSV + *S. pneumoniae*, **(C)** RSV + EF 1:200 + *S. pneumoniae*, and **(D)** RSV + EF 1:400 + *S. pneumoniae*. **(B)** Bar chart shows *S. pneumoniae* adhesion under different conditions: uninfected tissue (infected with *S. pneumoniae* but not RSV), RSV-infected, and RSV-infected tissues treated with Echinaforce^®^ (EF) at 1:200 and 1:400 dilutions. Data represent ALI-cultured EpiAirway tissues, with statistical significance indicated (*p < 0.05; **p < 0.01) ns = not significant.

Following apical infection with RSV, 3D pediatric EpiAirway tissues were treated on the basal side with EF extract at 1:200 and 1:400 dilutions for 72 h, after which the adherence of *S. pneumoniae* and Hib was assessed. RSV infection significantly enhanced the adhesion of *S. pneumoniae* by 8.8 ± 3.5-fold (*p* = 0.0016) compared to uninfected control tissues, which exhibited a low basal level of *S. pneumoniae* binding (Figure 1A). Notably, the formation of bacterial aggregates (biofilm-like structures) was evident on the surface of the virus-infected tissue (Figure 1B). Post-infection treatment with EF at dilutions of 1:200 and 1:400 reduced *S. pneumoniae* adhesion by 73% and 60%, respectively (Figures 1C,D), with the 1:200 dilution achieving statistical significance (*p* = 0.0003; Table 1).

**TABLE 1:** Analysis of RSV-induced bacterial adhesion and EF inhibition.

Condition	*S. pneumoniae* adhesion (fold change)	Hib adhesion (fold change)	% inhibition (*S. pneumoniae*)	% inhibition (Hib)
RSV	8.8 ± 3.5^(^**)	3.2 ± 1.1^(^**^)^	-	-
RSV + EF 1:200	1.78 ± 1.1^(^**)	0.93 ± 0.23^(^**^)^	80%	71%
RSV + EF 1:400	5.68 ± 2.6^(ns)^	2.84 ± 1.82^(ns)^	36%	11%

Quantification of *S. pneumoniae* and Hib adhesion to airway tissue following RSV infection, and the inhibitory effects of EF treatment at 1:200 and 1:400 dilutions. Fold changes are relative to uninfected controls; percentage inhibition is relative to RSV-infected controls. Data are presented as mean ± SD from three independent experiments (n = 3). Statistical significance: *p* < 0.01 (**), ns = not significant.

**FIGURE 2:**
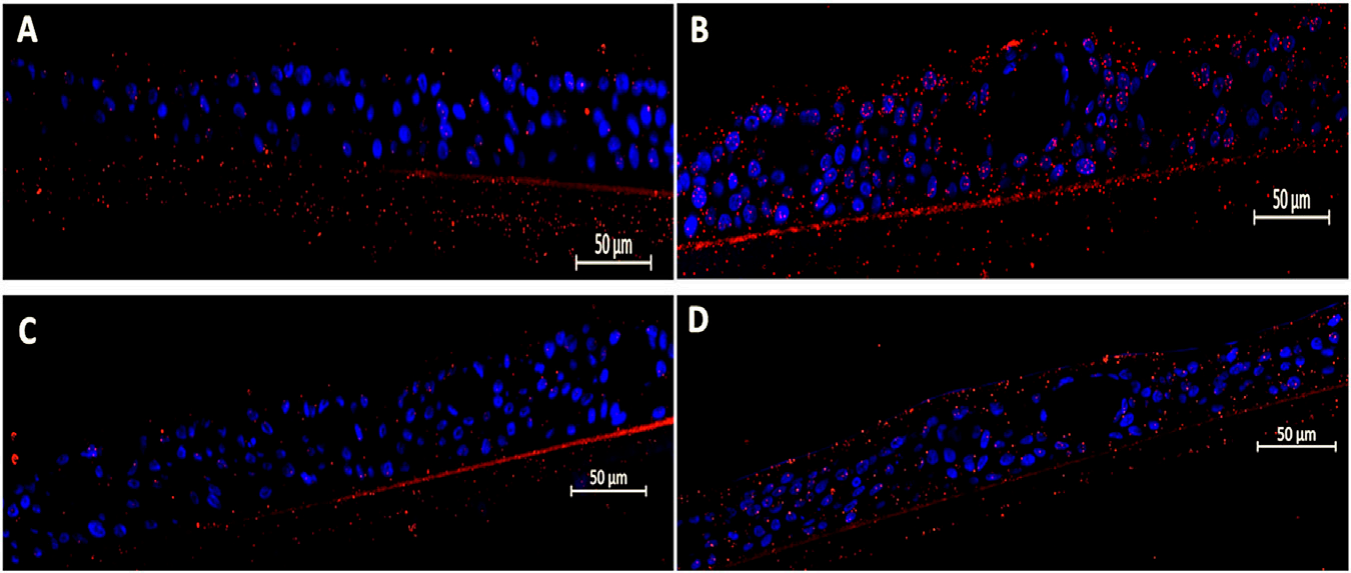
Efficacy of Echinaforce in reducing RSV-induced Hib adhesion in pediatric EpiAirway tissue. **(A)** EpiAirway tissues cultured in an air-liquid interface (ALI) were stained with anti-Hib antibody (green) and DAPI for nuclei, visualized at ×20 magnification. Representative images are shown for the following conditions: **(A)** Vehicle Control + Hib, **(B)** RSV + Hib, **(C)** RSV + EF 1:200 + Hib, and **(D)** RSV + EF 1:400 + Hib. **(B)** Bar chart shows Hib adhesion under different conditions: uninfected tissue (infected with Hib but not RSV), RSV-infected, and RSV-infected tissues treated with EF at 1:200 and 1:400 dilutions. Statistical significance is indicated *p* < 0.05 (*), ns = not significant.

Similarly, RSV infection led to a 3.2 ± 1.1-fold increase (*p* = 0.0042) in Hib adhesion compared to uninfected pediatric tissues, which visually appear to bind more Hib than *S. pneumoniae* endogenously (Figure 2A). Post-infection treatment with EF at a 1:200 dilution significantly reduced Hib adhesion by 71% (0.93 ± 0.23-fold, *p* = 0.0022), while the 1:400 dilution showed only a non-significant trend toward reduced adhesion (11% reduction, *p* = ns). In both cases, bacterial biofilm-like structures observed upon viral activation on the tissue apical side were completely resolved with EF treatment (Figures 2A–D, Table 1).

The results indicate that Hib showed a higher inherent binding to pediatric airway tissue compared to *S. pneumoniae*. RSV infection, increased adherence of *S. pneumoniae* by a factor of 8.8, and thus stronger than Hib by a factor of 3.2. This induction was potently reversed by treatment with EF extract, showing its ability to penetrate to epithelial tissue and become systemically active at the apical site (bioavailability). EF at dilutions of 1:200, corresponding to 80 μg/mL dry mass, almost completely prevented the virus-induced dysregulation to pre-infection levels.

### 3.3 Bacterial adhesion dysregulation in virus-infected NHBE cells

To further validate and extend findings, we studied different combinations of pathogenic partners in a more controlled environment, i.e., primary bronchial epithelial cells (NHBE), from the same pediatric donor. In particular, two groups of respiratory agents (enveloped vs non-enveloped viruses) were employed to discover any virus-specific dysregulation of bacterial binding, and whether this distinct regulation would be constituted through discrete bacterial adhesion receptors.

#### 3.3.1 *S. pneumoniae* adhesion

All viral stimuli, i.e., RSV, HPIV3, and RV14, significantly increased the adherence of *S. pneumoniae* 72 h post-infection in primary bronchial epithelial (NHBE) cells, whereby results from pediatric tissue were replicated (see Table 2; all p values <0.05). The strongest induction, by factor of 4.5 ± 1.5 and 7.7 ± 5.1, was observed for HPIV3 and RSV, respectively, both notorious agents of complicated infections during childhood. Under all conditions, EF treatment potently reduced (36%–76%) the increased bacterial binding and reversed dysregulation. Comparable with data from tissue, RSV infection resulted in a 4.5 ± 1.5-fold increase in *S. pneumoniae* adhesion (*p* = 0.0007), which was substantially reversed by EF at concentrations of 1:200 and 1:400 to 1.3 ± 0.7 (76% reduction, *p* = 0.0018) and 2.1 ± 0.9 (52% reduction, *p* = 0.011), respectively. HPIV3 infection led to a 7.7 ± 5.1-fold increase in *S. pneumoniae* adhesion, with EF at 1:200 concentration reducing to 3.7-fold, achieving a 59% reduction (*p* = 0.0166).

**TABLE 2:** Effect of EF on *S. pneumoniae* adhesion in virus-infected NHBE cells.

Condition	Adhesion fold change (mean ± SD)			% inhibition of adhesion		
	RSV	HPIV3	RV14	RSV	HPIV3	RV14
Virus + Vehicle	4.5 ± 1.5^(***)^	7.7 ± 5.1^(****)^	3.9 ± 1.64^(**)^	-	-	-
Virus + EF 1:200	1.3 ± 0.7^(**)^	3.7 ± 3.5^(*)^	1.5 ± 0.97^(***)^	76%	59%	61%
Virus + EF 1:400	2.1 ± 0.9^(*)^	6.6 ± 4.9^(*)^	2.5 ± 1.7^(*)^	52%	36%	37%

*S. pneumoniae* adhesion to NHBE cells infected with various respiratory viruses, expressed as a fold increase over uninfected cells, and the percentage inhibition by EF at 1:200 and 1:400 dilutions, calculated relative to virus-infected cells. Adhesion was quantified using colony-forming units (CFU), presented as fold increase and percent reduction**.** Statistical significance: *p* < 0.05 (*) *p* < 0.01 (**), p < 0.001 (***) and *p* < 0.0001 (****).

In contrast to enveloped viruses HPIV3 and RSV, the least induction of *S. pneumoniae* adhesion was observed for RV14 showing a 3.9-fold increase. Again, EF at 1:200 significantly reduced adhesion levels, and the effect for 1:400 dilution was seen as a trend that did not reach statistical significance (*p* > 0.05). With all tested viruses, EF extract reversed bacterial binding potently but never below the inherent binding levels found in absence of virus infection. EF therefore did not negatively influence the natural bacterial colonization in absence of infectious trigger.

#### 3.3.2 *Haemophilus influenzae* type b (Hib) adhesion

A similar, slightly more differentiated picture of induction was then found for Hib, withstrongest induction observed following RSV and HPIV3 (similar to *S. pneumoniae*). As shown in Table 3, RSV infection resulted in a 5.12-fold increase (**p < 0.01), HPIV3 infection led to a 9.2-fold increase (****p < 0.0001), with RV14 only marginally affecting Hib binding compared to uninfected controls.

**TABLE 3:** Effect of Echinaforce^®^ on Hib adhesion in virus-infected NHBE cells.

Condition	Adhesion fold change (mean ± SD)			% inhibition of adhesion		
	RSV	HPIV3	RV14	RSV	HPIV3	RV14
Viruses	5.12 ± 2.62^(**)^	9.2 ± 1.03^(****)^	1.8 ± 0.3^(**)^	-	-	-
Virus + EF 1:200	1.76 ± 0.86^(**)^	2.5 ± 0.27^(****)^	0.55 ± 0.7^(***)^	65%	73%	69%
Virus + EF 1:400	2.1 ± 1.05^(*)^	3.6 ± 0.32^(****)^	0.8 ± 0.05^(*)^	58%	60%	55%

Hib adhesion to NHBE cells infected with various respiratory viruses, expressed as a fold increase over the control (uninfected cells), and the percentage inhibition of this adhesion by EF extract at dilutions of 1:200 and 1:400. Quantification was performed using colony-forming units (CFU). Data represent mean ± SD from three independent experiments (*n* = 3). Statistical significance: *p* < 0.05 (*), p < 0.01 (**), p < 0.001 (***), *p* < 0.0001 (****), ns = not significant.”

In RSV-infected cells, EF at 1:200 reduced adhesion by 65% (1.76-fold, **p < 0.01), and at 1:400 by 58% (2.1-fold, *p < 0.05). For HPIV3-infected cells, EF at 1:200 resulted in a 73% reduction in adhesion (2.5-fold), while at 1:400, the reduction was 60% (3.6-fold). With RV14, EF at 1:200 reduced adhesion by 69% (***p < 0.001), and at 1:400, the reduction was 55% (0.8-fold, *p < 0.05).

EF showed substantial and broad reductions of induced Hib adhesion to pediatric epithelial cells upon infection with different respiratory viruses. Similar to findings from pediatric tissue, endogenous Hib binding was stronger than for *S pneumoniae* whereas respiratory virus infections further accentuated this imbalance, which was reversed under EF treatment. In NHBE cells, the endogenous binding of Hib was (50 ± 10-fold) higher than that of *S. pneumoniae*, aligning with findings from pediatric tissue (20 ± 4-fold).

### 3.4 Antibody-mediated receptor blocking to reduce bacterial adhesions

#### 3.4.1 Blocking *S. pneumoniae* adhesion to virus-infected NHBE cells

**FIGURE 3:**
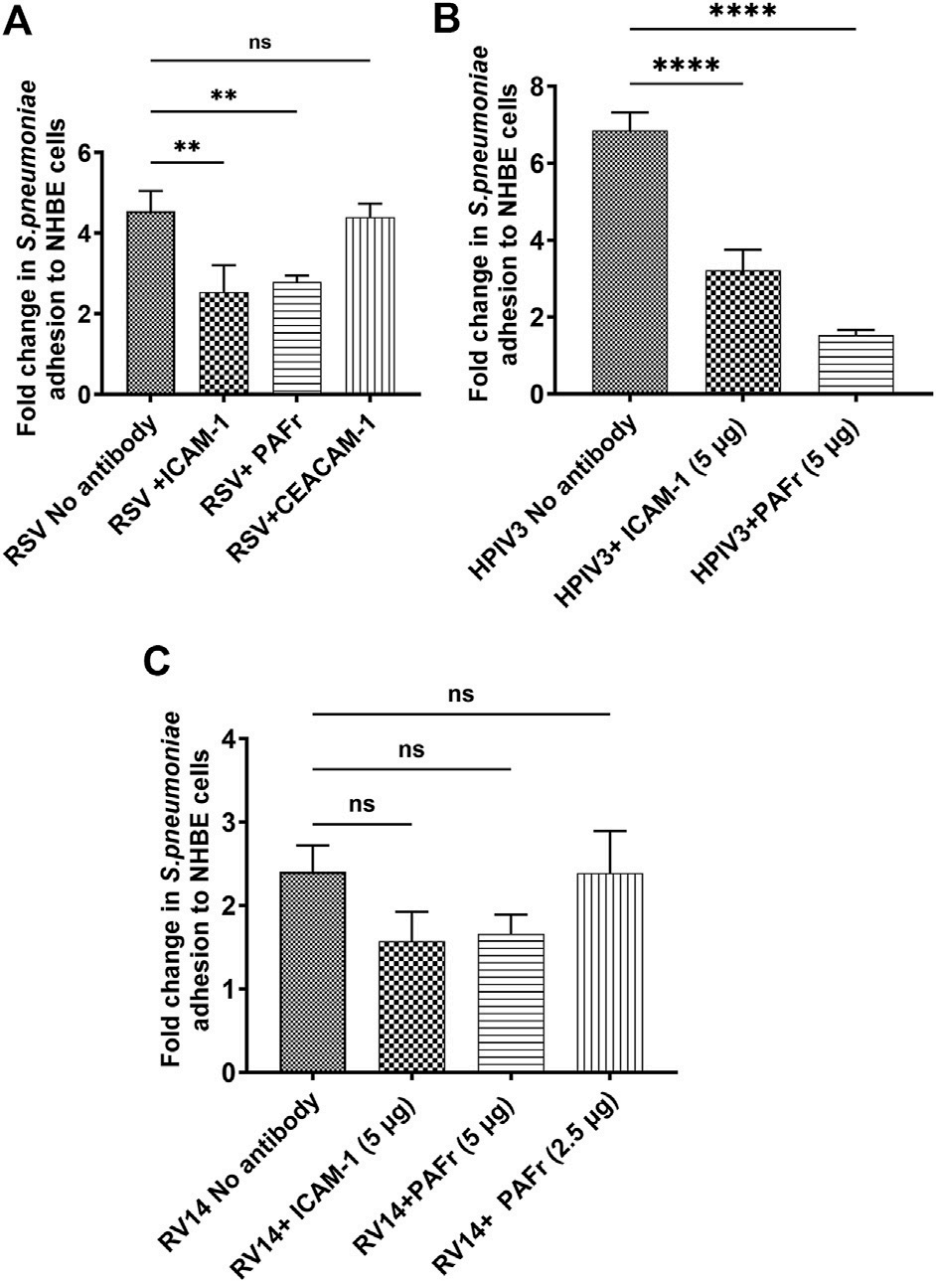
Blocking ICAM-1 and PAFr reduces *S. pneumoniae* adhesion to virus-infected NHBE cells. Anti-ICAM-1 and anti-PAFr antibodies significantly reduced *S. pneumoniae* adhesion in **(A)** RSV- and **(B)** HPIV3-infected NHBE cells, rather than with PV14 infection **(C)**. Results are expressed as mean values ±SD from three independent experiments Statistical significance: *p* < 0.01 (**), *p* < 0.0001 (****), “ns” indicates no significance.

As previously shown, dysregulated bacterial colonization may result from specific expression patterns of bacteria-binding receptors (e.g., ICAM-1, PAFr, CEACAM-1) whereas blocking antibodies serve as a useful tool to study individual receptor’s contribution to the overall affinity (Vimalanathan et al., 2017). Virus-infected cells were treated with blocking antibodies against the above-mentioned receptors at a concentration of 5 μg/mL for 1 hour before inoculating *S*. *pneumoniae* binding. Blocking ICAM-1 and PAFr significantly reduced adherence upon RSV and HPIV3 infection to pre-infection levels of between 1 or 2-fold induction (Figures 3A,B, **p < 0.01). This was particularly evident in HPIV3 infections, where PAFr played a key role in binding *S. pneumoniae*. Consistent with previous research, CEACAM-1 played a subordinate role with *S. pneumoniae* binding and the antibody did not significantly impact bacterial adhesion, which could be compensated by the presence of upregulated ICAM-1 and PAFr. For RV14 infection, treatment with ICAM-1 and PAFr antibodies resulted in a slight decrease in bacterial adhesion, although these changes were not statistically significant (Figure 3C). This may be due to the generally low induction of *S. pneumoniae* by RV14, lower endogenous binding, and thus a limited possibility of reversion, whereas the importance of ICAM-1 and PAFr as binding receptors for *S. pneumoniae* was primarily demonstrated upon RSV and HPIV3 induction.

#### 3.4.2 Blocking Hib adhesion to virus infected NHBE cells

**FIGURE 4:**
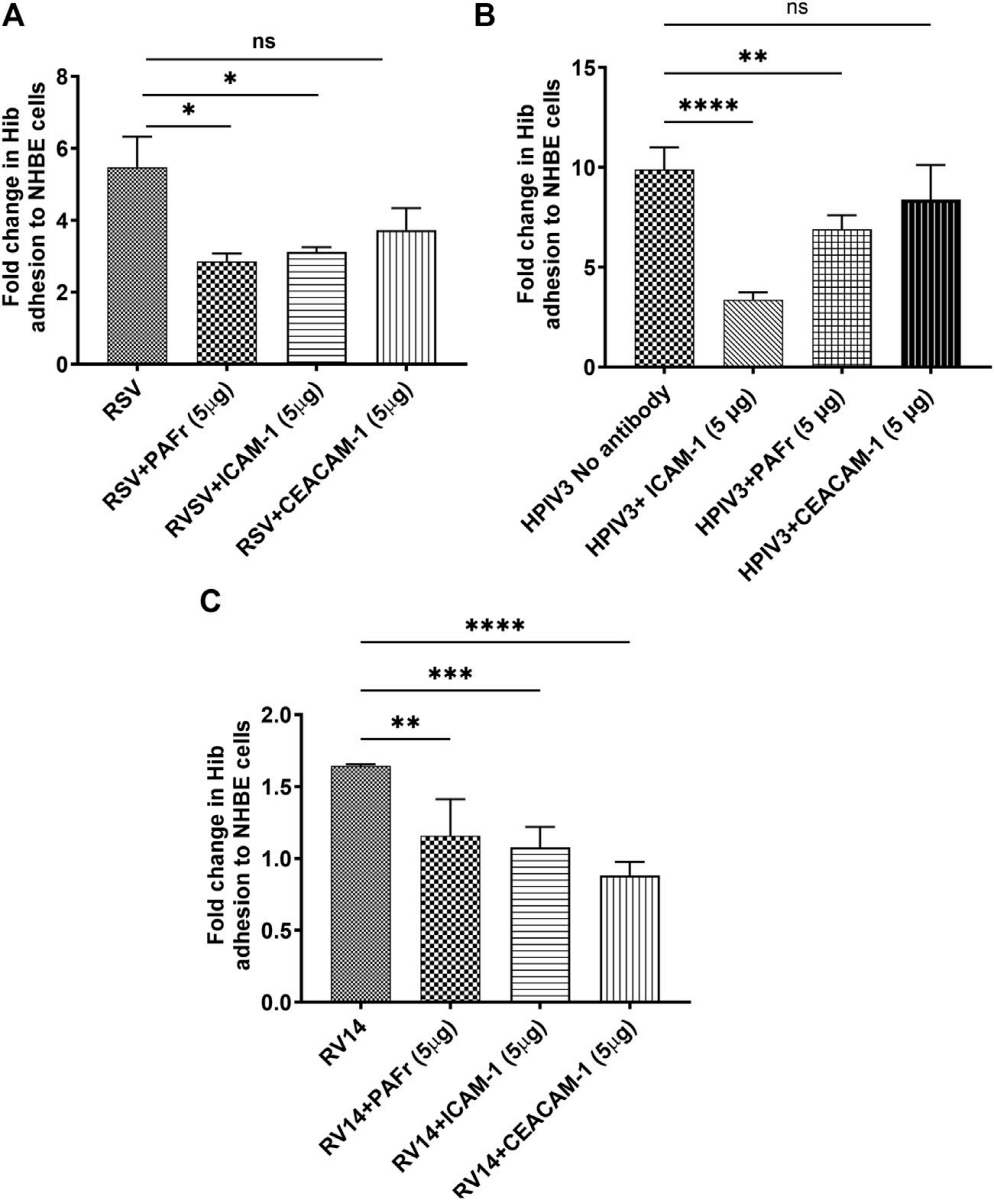
Blocking ICAM-1, PAFr, and CEACAM-1 reduces Hib adhesion to virus-infected NHBE cells. **(A)** Blocking ICAM-1 and PAFr significantly reduced Hib adhesion at all viral stimuli (*p* < 0.05 (*) to *p* < 0.0001 (****)). **(B)** ICAM-1 blocking was most effective in HPIV3-infections. **(C)** CEACAM-1 blocking was most effective with RV14 *p* < 0.0001 (****). Data are presented as mean ± SD from three independent experiments; “ns” denotes no significant difference.

The same blocking antibodies were used to identify Hib binding receptors as above. In RSV-infected cells, anti-PAFr antibody resulted in a notable reduction in Hib adhesion (*p < 0.05), similar to anti-ICAM-1 antibody. Anti-CEACAM-1 antibody did not show a significant binding reduction, which seems primarily mediated through PAFr and ICAM-1 receptors (Figure 4A). A lower binding affinity to CEACAM-1 can thus be deduced.

A substantial increase in Hib adhesion (9.3-fold) was observed in HPIV3-infected cells. Blocking ICAM-1 resulted in the most significant reduction in adhesion (****p < 0.0001), highlighting its dominant role in Hib binding during HPIV3 infections. Blocking PAFr also significantly reduced adhesion, though its effect was slightly less pronounced compared to ICAM-1 (***p < 0.001). Interestingly, CEACAM-1 blockade had no significant impact on adhesion in HPIV3-infected cells, confirming that Hib binding is predominantly mediated by ICAM-1 and PAFr under these conditions (Figure 4B).

Although RV14 only slightly increased Hib adhesion, blocking antibodies against PAFr, ICAM-1, and CEACAM-1 all led to significant reductions in adhesion (**p < 0.01, ***p < 0.001, ****p < 0.0001), with varying effect sizes. Notably, CEACAM-1 antibody treatment led to the greatest reduction in adhesion, suggesting a more prominent role for CEACAM-1 in mediating Hib adhesion during RV14 infection. (Figure 4C).

Overall, the adhesion of Hib to NHBE cells is mediated by specific receptors, which depends on the infecting virus. In RSV-infected cells, Hib adhesion is primarily driven by both, PAFr and ICAM-1, ICAM-1 is particularly important with HPIV3 infections, whereas CEACAM-1 had no notable impact. With RV14 infections, all three receptors (PAFr, ICAM-1, and CEACAM-1) contributed to Hib adhesion, with CEACAM-1 playing a prominent role in bacterial adhesion.

### 3.5 Effect of EF on virus-induced bacterial receptors (PAFr, ICAM-1 and CEACAM-1)

As demonstrated above, bacteria binding is directed through the presence and regulation of binding receptors, with different pathogens employing distinct sets of ligands for adhesion. Next, we studied whether above-mentioned viruses would be able to induce characteristic patterns of binding receptors entailing differential bacterial regulation. Alongside effects of Echinacea extracts on those expression patterns were studied.

#### 3.5.1 PAFr expression

##### 3.5.1.1 Virus-induced PAFr expression in NHBE cells

**FIGURE 5:**
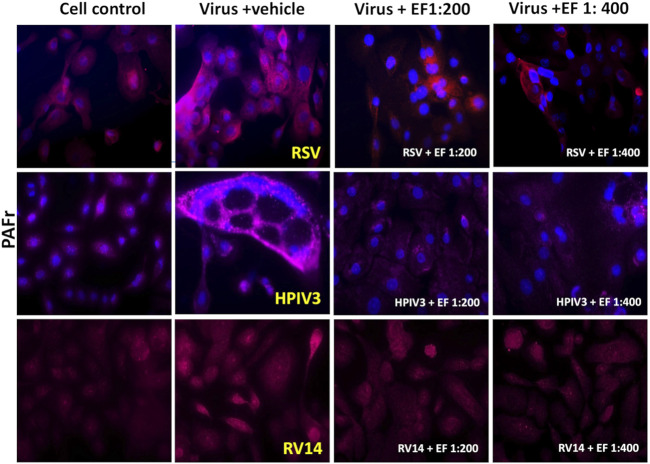
The Effect of EF extract on infection-induced PAFr expression in NHBE cells. PAFr expression in NHBE cells following infection with RSV, HPIV3 and RV14, 72 h post-infection, with or without EF treatment. This Figure represents one of three independent experiments, captured at ×20 magnification.

First, a significant increase in PAFr expression was observed, a receptor for both *S. pneumoniae* and Hib, following HPIV3 and RSV infection, showing a 2.4 ± 1.53 up to 3.25 ± 2-fold enhancement, respectively (both *p < 0.05, Table 4). In Figure 5, apparent formation of syncytia (polynuclear cell fusions) secondary to HPIV3 infections were detected with high-dense expression of PAFr. These might explain the tremendous effects in PAFr blocking experiments with HPIV3 infections (e.g., with *S. pneumonia*e). Treatment with EF at dilutions of 1:200 and 1:400 reduced PAFr expression back to non-infection levels of lower than 1.5-fold as based on statistical significance (Figure 5; Table 4). EF-effects were most pronounced at sites of syncytia formation.

**TABLE 4:** Quantification of virus-induced PAFr expression in 3D NHBE cells.

Virus-augmented PAFr (Fold Increase/Cell Control)			
Condition	RSV (P-value)	HPIV3 (P-value)	RV14 (P-value)
Viruses	3.25 ± 2 (****)	2.4 ± 1.53 (*)	1.75 ± 1.14 (*)
Virus + EF 1:200	1.5 ± 0.76 (**)	1.1 ± 1.04 (*)	1.53 ± 0.24 (ns)
Virus + EF 1:400	1.27 ± 0.91 (***)	1.3 ± 0.60 (*)	1.58 ± 0.34 (ns)

The table presents fold increases in PAFr expression following infection with RSV, HPIV3, and RV14, as well as the effects of EF treatment at 1:200 and 1:400 dilutions. Values are shown as fold increases (mean ± SD) with corresponding p-values. Significant differences are indicated as follows: *p* < 0.05 (*), *p* < 0.01 (**), *p* < 0.001 (***), *p* < 0.0001 (****), ns denotes not significant.

PAFr induction through RV14 was low (induction by 1.75 ± 1.14) but still significant on the level of alpha <0.05. Based on the higher affinity of *S*. *pneumoniae* to PAFr, compared than ICAM-1 (as observed in blocking experiments) or Hib, this could serve as an explanation for preferred binding of the prior during RV14 infections. In this case, the EF extract showed negligible effects and did not further suppress or impact the natural colonization of bacteria in absence of viral induction.

In conclusion, the increase in PAFr expression was most pronounced with the enveloped viruses RSV and HPIV3 and less so with RV14 infection, which corresponds very well with bacterial dysregulation experiments as shown above. EF treatment, particularly at 1:200 and to a lesser extend at the higher dilution of 1:400, effectively reversed any induced PAFr expression, with the most significant reductions observed in RSV and HPIV3 infections.

#### 3.5.2 ICAM-1 expression

##### 3.5.2.1 Virus-induced ICAM-1 expression in NHBE cells

**FIGURE 6:**
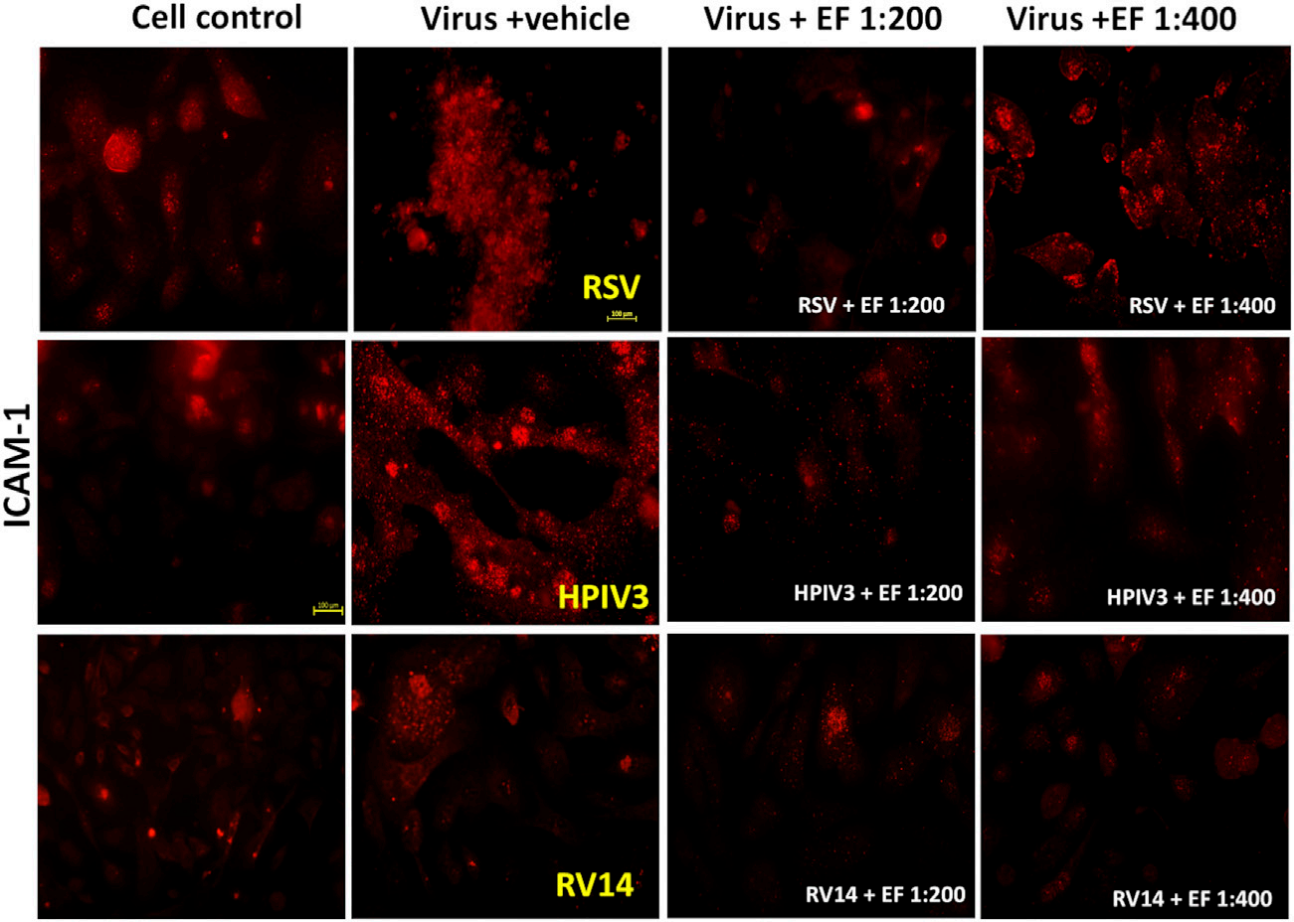
Effect of EF extract on induced ICAM-1 expression in NHBE cells. The image represents one experiment out of three independent experiments, captured at ×20 magnification.

Figure 6 and Table 5 reveal, how RSV and HPIV3 infection significantly increased ICAM-1 expression by 4.7 ± 3.47-fold (*p < 0.05) and by 5.0 ± 1.31-fold, respectively (**p < 0.01). In contrast, RV14 infections resulted in non-significant increases in ICAM-1 expression, with fold changes of only 1.32 ± 1.24. As for PAFr, results nicely comply with earlier observations of low bacteria-adhesion propensity of non-enveloped rhinoviruses in comparison with enveloped viruses and inability to reverse binding through blocking ICAM-1 antibodies in the prior case.

**TABLE 5:** Quantification of virus-induced ICAM-1 expression.

Virus-Augmented ICAM-1 (Fold Increase/Cell Control)			
Condition	RSV (P-value)	HPIV3 (P-value)	RV14 (P-value)
Viruses	4.7 ± 3.47 (*)	5.0 ± 1.31 (**)	1.32 ± 1.24 (ns)
Virus + EF 1:200	1.3 ± 0.88 (*)	1.5 ± 0.53 (***)	0.49 ± 0.39 (ns)
Virus + EF 1:400	1.64 ± 1.25 (ns)	3.5 ± 2.32 (ns)	0.87 ± 0.35 (ns)

The table shows fold increases in ICAM-1 expression after RSV, HPIV3, and RV14 infection, and the effects of EF at 1:200 and 1:400 dilutions. Values are fold increases with mean ± SD and p-values. Significant differences: *p* < 0.05 (*), p < 0.01 (**), p < 0.001 (***), *p* < 0.0001 (****), ns = not significant.

##### 3.5.2.2 Effect of EF on ICAM-1 expression

Treatment with EF at a dilution of 1:200 reduced ICAM-1 expression in RSV-infected cells to 1.3 ± 0.88-fold (*p < 0.05), indicating a significant inhibition for the higher and a trend for lower concentrations of 1:400. For HPIV3-infected cells, EF treatment at 1:200 led to a reduction in ICAM-1 expression to 1.5 ± 0.53-fold (***p < 0.001). However, in RV14-infected cells, EF treatment at 1:200 did not significantly alter ICAM-1 expression, with fold changes of 1.53 ± 0.58 and 0.49 ± 0.39, respectively (Table 5). Again, this might be explained by insufficient induction by rhinovirus that cannot be significantly reversed by EF although trends were observed.

#### 3.5.3 CEACAM-1 expression

##### 3.5.3.1 Virus-induced CEACAM-1 expression in NHBE cells

RSV infection led to a significant upregulation of CEACAM-1 by 12 ± 7.3-fold (****p < 0.0001), whereas HPIV3 caused a 7.3 ± 3.8-fold increase (**p < 0.01). In contrast to ICAM-1 or PAFr, CEACAM-1 was upregulated by RV14 resulting in 5.79 ± 4.05-fold increases (****p < 0.0001), respectively. Blocking experiments implicated that CEACAM-1 owns a negligible role in binding of *S. pneumoniae*. Despite considerable CEACAM-1 induction through RV, Hib binding was still not strongly induced. This might be consequence of concomitant low ICAM-1/PAFr induction by RV and/or an overall low affinity of CEACAM-1 for Hib. Again, prominent formation of syncytia with dense CEACAM-1 expression on cells was found.

##### 3.5.3.2 Effect of EF on CEACAM-1 expression

**FIGURE 7:**
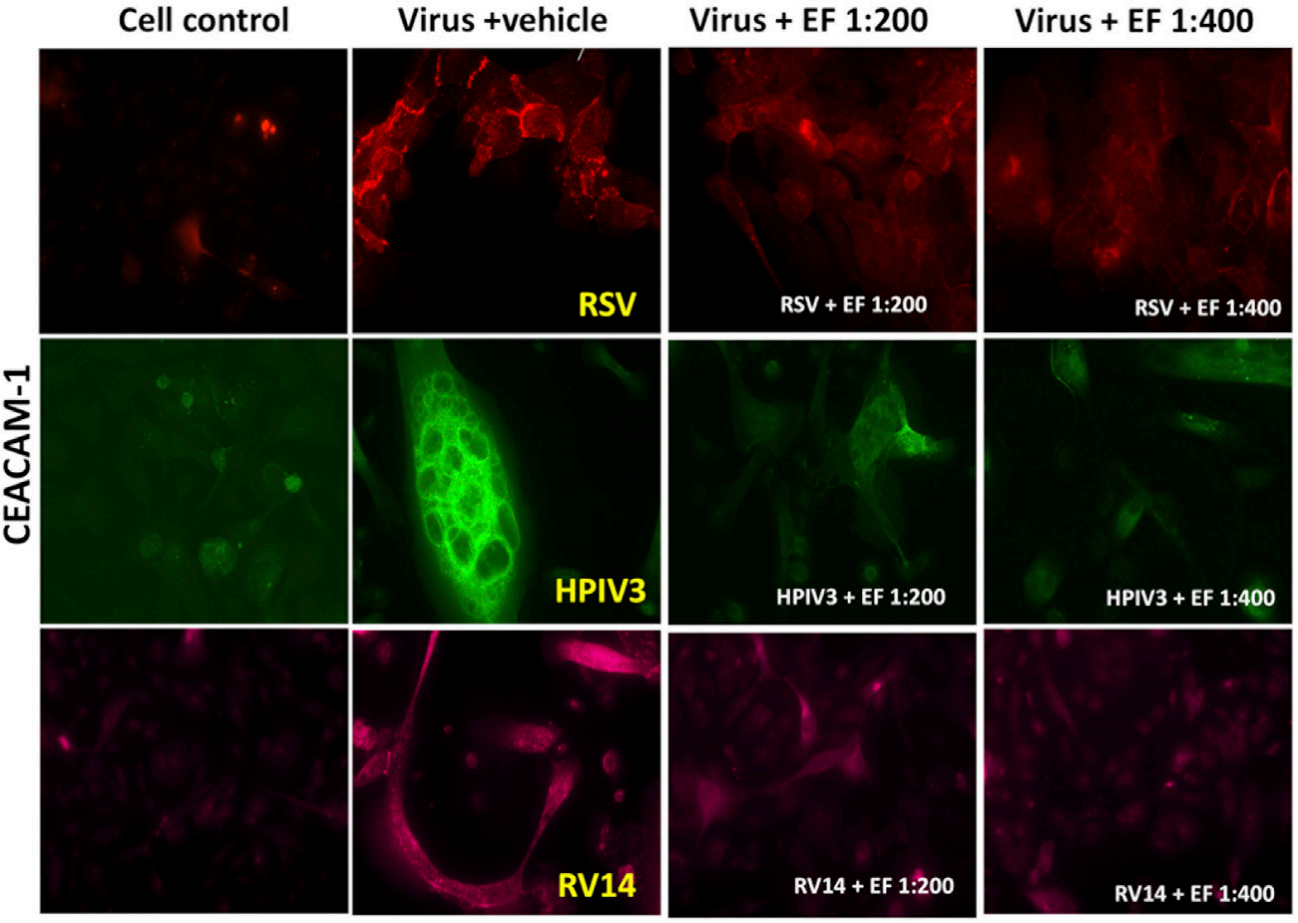
Effects of EF extract on CEACAM-1 expression in NHBE cells. The results are from one representative experiment, typical of three independent experiments, viewed at ×20 magnification. Fluorescence intensity was measured using ImageJ.

Treatment with EF at a 1:200 dilution significantly reduced CEACAM-1 expression in RSV-infected cells to 6.7 ± 5.07-fold (*p < 0.05) and in RV14-infected cells to 1.78 ± 1.2-fold **(*p* < 0.01). Notably, in HPIV3-infected cells, EF at 1:200 drastically reduced CEACAM-1 expression to 0.5 ± 0.45-fold (***p < 0.001). When treated with EF at a 1:400 dilution, the reductions were less pronounced. RSV-induced expression was reduced to 8.6 ± 7.4-fold, HPIV3-induced expression to 3.5 ± 2.32-fold, RV14-induced expression to 1.8 ± 0.88-fold, with significant reduction observed only in RV14-infected cells (**p < 0.01) (Table 6). These findings suggest that EF, particularly at the 1:200, effectively reduces CEACAM-1 expression in RSV, HPIV3, and RV14 infections (Figure 7).

**TABLE 6:** Quantification of virus-induced CEACAM-1 expression in NHBE cells.

Virus augmented CEACAM-1 (Fold change/cell control)			
Condition	RSV (P-value)	HPIV3 (P-value)	RV14 (P-value)
Viruses	12 ± 7.3 (****)	7.3 ± 2.8 (**)	5.79 ± 4.05 (****)
Virus + EF 1:200	6.7 ± 5.07 (*)	0.5 ± 0.45 (***)	1.78 ± 1.2 (**)
Virus + EF 1:400	8.6 ± 7.4 (ns)	3.5 ± 2.32 (ns)	1.8 ± 0.88 (**)

This table presents the fold increase in CEACAM-1 expression following infection with RSV, HPIV3, and RV14, and the effects of EF treatment at 1:200 and 1:400 dilutions. Values are shown as fold increases relative to control, with mean ± SD and p-values. Significant differences: *p* < 0.05 (*), p < 0.01 (**), p < 0.001 (***), *p* < 0.0001 (****), ns = not significant.

Finally, above results obtained from cells could be reproduced in organotypic tissues, with RSV enhancing the expression of PAFr by 4.1 ± 1.4 -fold, CEACAM-1 by 1.5 ± 0.7-fold, and ICAM-1 by 2.6 ± 0.5-fold (all *p < 0.05). Treatment with EF at a 1:200 concentration strongly inhibited this upregulation, reducing PAFr, CEACAM-1, and ICAM-1 expression by 88%, 80%, and 88%, respectively. At a lower concentration (1:400), the inhibition is weaker, with 63% for PAFr, 73% for CEACAM-1, and only 35% for ICAM-1, indicating a dose-dependent effect.

## 4 Discussion

Respiratory viruses are common triggers of respiratory tract infections (RTIs) but differ significantly in their potential to cause bacterial superinfections and associated complications (van Benten et al., 2003). While rhinoviruses can cause significant inflammation, they generally result in less tissue damage and fewer bacterial superinfections compared to RSV or HPIV3. Severe pathology caused by rhinoviruses is typically limited to high-risk individuals, such as those with asthma or immunocompromised conditions (Kennedy et al., 2012; Vandini et al., 2019). In contrast, enveloped viruses like RSV and HPIV3 often induce extensive cellular damage or syncytia formation, which promotes bacterial colonization, particularly through the upregulation of bacteria-binding receptors (Villenave et al., 2011; Hanish et al., 2023). Understanding these mechanisms is crucial for developing interventions, as demonstrated in our study using an *ex vivo* reconstituted bronchial tissue model derived from a 6-year-old child.

We observed a greater basal propensity for Hib binding compared to *S. pneumoniae* in pediatric bronchial tissue in the absence of viral stimuli. However, RSV infection enhanced *S. pneumoniae* binding more substantially than Hib, resulting in pronounced bacterial dysregulation. EF extract potently reversed these effects, reducing bacterial aggregation and resolving biofilm formation by both *S. pneumoniae* and Hib on the apical side. This systemic pharmacological activity may explain Echinacea’s potential to mitigate bacterial superinfections induced by viral RTIs.

Expanding our research to other respiratory viruses in primary bronchial epithelial cells derived from the same 6-year-old donor, revealed that both, RSV and HPIV3 significantly increased the binding of *S*. *pneumoniae* and Hib, by factors of 4.5–7.7, compared to 1.8 to 3.9 for rhinoviruses. This finding underscores HPIV3 as another key inducer of bacterial co-infections, corroborating clinical observations by Johnson et al. (2008) (Johnson et al., 2008), RSV and HPIV3 facilitated bacterial adhesion primarily through overexpression of ICAM-1 and PAFr, while CEACAM-1 played a minor role in Hib binding and was irrelevant for *S. pneumoniae* adherence. Rhinovirus predominantly induced CEACAM-1 without significant upregulation of high-affinity receptors (ICAM-1 or PAFr), resulting in marginal bacterial binding. These findings reflect the diverse mechanisms by which respiratory viruses interact with bacterial pathogens, highlighting the complexity of receptor regulation and its critical role in superinfections. Moreover, increased bacterial adhesion may be mediated not only by virus infection but also by pro-inflammatory cytokines such as IL-6, IL-1α, and TNF-α, which upregulate receptors like ICAM-1, PAFr, and CEACAM1. These cytokines are commonly secreted by RSV-infected airway epithelium and have also been observed in infections with HPIV3, influenza, and rhinovirus (Patel et al., 1995; Chang et al., 2003; Avadhanula et al., 2007; Hu et al., 2022).

HPIV3 uniquely formed giant syncytia, characterized by dense PAFr (Figure 5) and CEACAM-1 (Figure 7) expression on the membrane, creating hotspots for enhanced bacterial adherence. Previous studies have reported that HPIV3 and RSV can upregulate ICAM-1 and CEACAM-1 expression in NHBE cells (Avadhanula et al., 2006). However, our study is the first to also report PAFr upregulation specifically on the membrane of NHBE cells and on the tissue model. These findings provide further evidence of (enveloped) viruses’ capacity to employ multiple strategies, including syncytia formation and receptor upregulation, to enhance bacterial adhesion, demonstrating its unique role in viral-bacterial co-infections.

Despite the complex pathways underlying virus-induced bacterial dysregulation, EF extract demonstrated a remarkable ability to reverse these pathogenic effects. Although applied to the basal side, it permeated to become bioavailable on the apical side of the epithelium. It restored bacterial adhesion to pre-infection levels without suppressing the endogenous binding and without inducing dysregulation. This dual action—modulating receptor induction and restoring bacterial homeostasis—exemplifies EF extract’s potential as a broadly applicable therapeutic intervention for managing viral-bacterial co-infections.

Importantly, our findings are consistent with clinical observations. A meta-analysis by Gancitano et al. reported a 32% reduction in viral RTIs and up to a 56% reduction in bacterial superinfections with Echinacea supplementation (RR = 0.44; 95% CI 0.36–0.54) (Gancitano et al., 2024). Antibiotic use was thereby reduced by 40%–71%, with the most pronounced benefits observed in children, where antibiotic treatment days decreased by up to 80% (Ogal et al., 2021). These clinical outcomes correspond with EF’s ability in maintaining bacterial homeostasis through binding receptor regulation, suggesting a plausible mechanism for its role in mitigating RTI complications.

Our study has limitations. It was essentially confined to an *ex vivo* test system using only two bacterial strains and three respiratory viruses, limiting its scope in capturing the diversity of viral-bacterial interactions. However, the use of organotypic pediatric tissue (ALI technology), clinical bacterial isolates (*S. pneumoniae* and Hib), and key pediatric viral pathogens (RV, RSV, and HPIV3) ensured a model closely mirroring *in vivo* conditions. Echinacea was applied to the basal side of the epithelium to assess its capacity to penetrate the tissue. The pharmacokinetics of herbal extracts is highly complex and depends on the bioavailability of multiple phytochemical substances. Alkylamides are important marker compounds involved in antiviral and immune-modulatory actions, the physiological relevance of which was demonstrated in CaCO2 cells and upon oral administration of EF extract (Matthias et al., 2005; Woelkart et al., 2006). In this context, clinical studies and meta-analyses (Gancitano et al., 2024) provide strong evidence for the *in vivo* relevance of Echinacea’s actions as described, in this work.

Interestingly, Echinacea’s effect in preventing bacterial superinfections and thus reducing the need for antibiotics was most pronounced in the pediatric population under 12 years of age. This was observed using a mixed dosage regimen consisting of a basic prevention dose of 1,200 mg EF extract with increased doses of up to 2,000 mg EF extract during acute respiratory illnesses (Ogal et al., 2021). This corresponds to approximately half the dosage recommended for adolescents and adults, for whom studies have employed similar staged dosing (Jawad et al., 2012). The inclusion of both enveloped and non-enveloped viruses allowed us to explore the pathological diversity of infections. SARS-CoV-2 was also studied; however, the results have not been included in this manuscript as further analysis is ongoing.

Overall, these results provide new insights into the pathogenesis of viral-bacterial co-infections in children and highlight the dual action of EF extract in preventing bacterial dysbiosis and biofilm-like formation, particularly in complex pathogenic combinations such as HPIV3 and *S. pneumoniae*. This study also reinforces the value of ALI culture systems in advancing our understanding of respiratory infections and in developing targeted therapeutic strategies, such as Echinacea. Considering the importance of viral respiratory tract infections and their associated health consequences, including increased antibiotic use, further large-scale clinical studies are warranted to confirm these benefits under real-world conditions, especially in children.

## 5 Conclusion

Respiratory viruses, such as RSV and HPIV3, induce bacterial binding to host tissue by upregulating specific bacteria-binding receptors, including PAFr, ICAM-1, and CEACAM-1. This receptor upregulation facilitates increased adhesion and biofilm-like formation by *S.pneumoniae* and Hib, thereby elevating the risk of secondary infections and related complications. These effects were more pronounced with RSV and HPIV3 compared to RV, highlighting virus-specific mechanisms that contribute to disease severity.

Our findings demonstrate that *E. purpurea* (Echinaforce^®^) effectively restores bacterial homeostasis by downregulating virus-induced receptor expression and inhibiting biofilm-like formation on the airway epithelium. Importantly, EF treatment did not affect the natural, baseline level of bacterial colonization in uninfected tissues, indicating a selective action that is activated under pathogenic conditions.

This dual pharmacological activity, combining antiviral effects with the prevention of bacterial overgrowth, provides a mechanistic basis for the clinical benefits observed with Echinacea purpurea, including reduced respiratory tract infection (RTI) complications and decreased antibiotic use, particularly in pediatric populations. These findings support and suggest that Echinaforce^®^ could contribute to reducing the risk of bacterial superinfections during respiratory viral illnesses, with potential relevance to pediatric populations.

## Data Availability

The datasets presented in this study can be found in online repositories. The names of the repository/repositories and accession number(s) can be found below: https://data.mendeley.com/preview/mh7tjsgzsn?a=82c734c8-1a16-4805-89a4-f5080f8945be.
