# Correction: Respiratory virus-induced bacterial dysregulation in pediatric airway tissue and the dual actions of Echinacea in reducing complications

**DOI:** 10.3389/fphar.2025.1656368

**Published:** 2025-08-07

**Authors:** Selvarani Vimalanathan, Mahfuza Sreya, Ranganayaki Nandanavanam, Roland Schoop, Giuseppe Gancitano, Saba Saberi, Anna Malikovskaia, James Hudson

**Affiliations:** ^1^ Pathology and Laboratory Medicine, University of British Columbia, Vancouver, BC, Canada; ^2^ Women+ and Children’s Health Sciences, University of British Columbia, Vancouver, BC, Canada; ^3^ Applied Biology, University of British Columbia, Vancouver, BC, Canada; ^4^ Medical Department, A.Vogel AG, Roggwil, Switzerland; ^5^ Department of Experimental and Clinical Medicine, University of Florence, Florence, Italy

**Keywords:** pediatrics, EpiAirway viral-bacterial superinfections, *Streptococcus pneumoniae*, *Haemophilus influenzae* type b, respiratory syncytial virus, human parainfluenza virus type 3, rhinovirus, *Echinacea purpurea*

In the published article, there were errors in Figures 1, 2 as published. The figures were truncated on one side, displaying only part of the images. The corrected Figures 1, 2 and their captions appear below.

**FIGURE 1:**
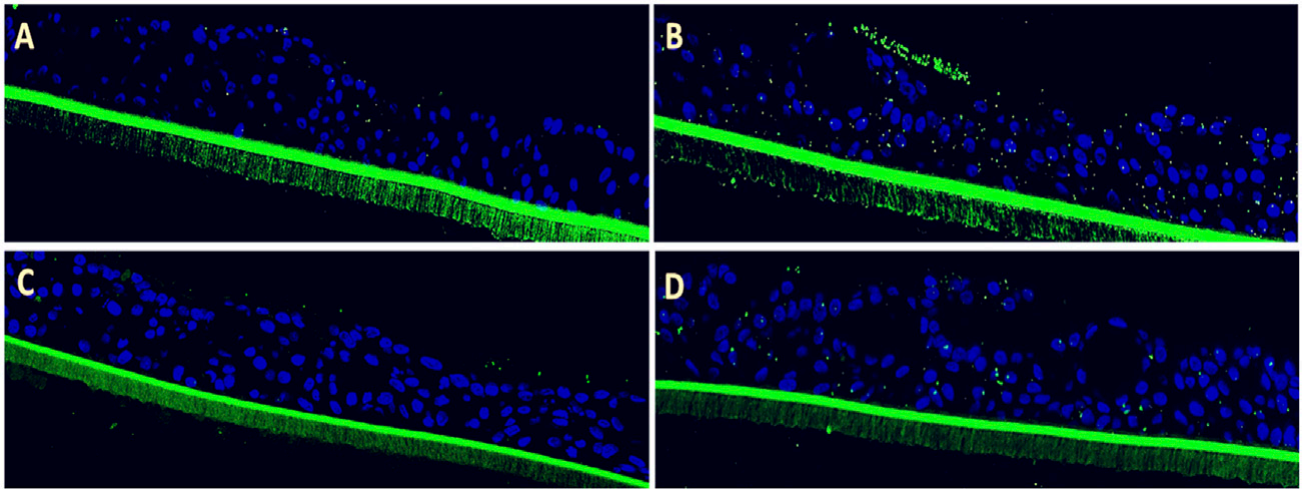
Efficacy of Echinaforce in reducing RSV-induced *S. pneumoniae* adhesion in pediatric EpiAirway tissue. **(A)** EpiAirway tissues cultured in an air-liquid interface (ALI) were stained with anti-*S. pneumoniae* antibody (green) and DAPI for nuclei, visualized at ×20 magnification. Representative images are shown for the following conditions: **(A)** Vehicle Control + *S. pneumoniae*, **(B)** RSV + *S. pneumoniae*, **(C)** RSV + EF 1:200 + *S. pneumoniae*, and **(D)** RSV + EF 1:400 + *S. pneumoniae*. **(B)** Bar chart shows *S. pneumoniae* adhesion under different conditions: uninfected tissue (infected with *S. pneumoniae* but not RSV), RSV-infected, and RSV-infected tissues treated with Echinaforce^®^ (EF) at 1:200 and 1:400 dilutions. Data represent ALI-cultured EpiAirway tissues, with statistical significance indicated (*p < 0.05; **p < 0.01) ns = not significant.

**FIGURE 2:**
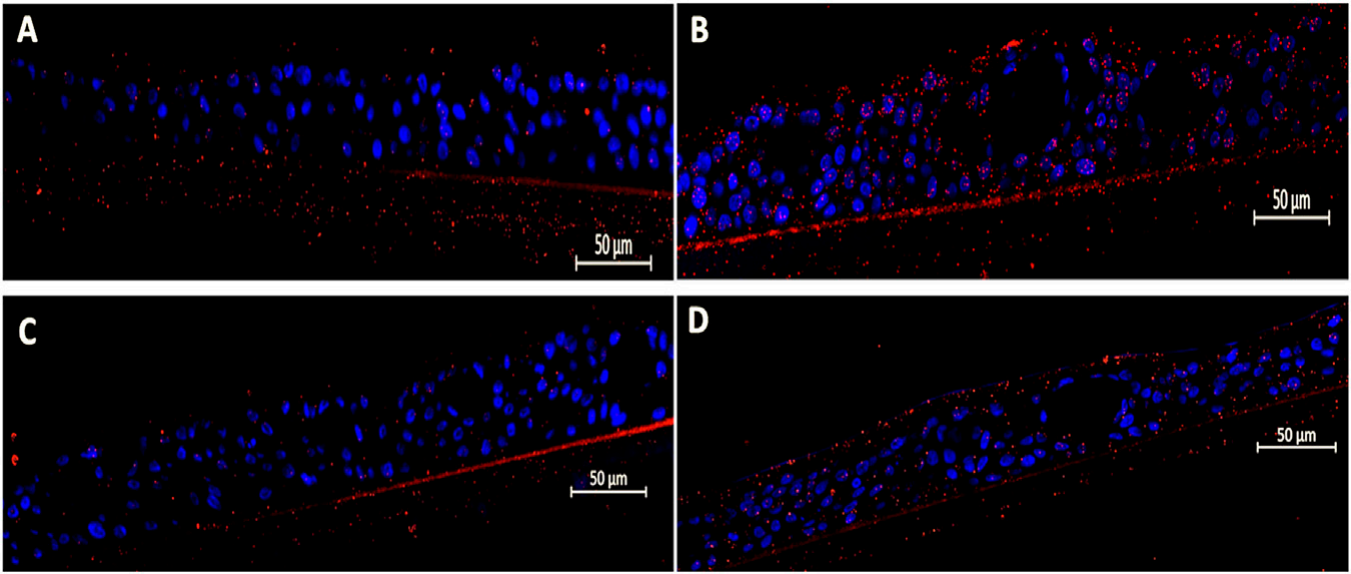
Efficacy of Echinaforce in reducing RSV-induced Hib adhesion in pediatric EpiAirway tissue. **(A)** EpiAirway tissues cultured in an air-liquid interface (ALI) were stained with anti-Hib antibody (green) and DAPI for nuclei, visualized at ×20 magnification. Representative images are shown for the following conditions: **(A)** Vehicle Control + Hib, **(B)** RSV + Hib, **(C)** RSV + EF 1:200 + Hib, and **(D)** RSV + EF 1:400 + Hib. **(B)** Bar chart shows Hib adhesion under different conditions: uninfected tissue (infected with Hib but not RSV), RSV-infected, and RSV-infected tissues treated with EF at 1:200 and 1:400 dilutions. Statistical significance is indicated *p* < 0.05 (*), ns = not significant.

The original version of this article has been updated.

